# Public health round-up

**DOI:** 10.2471/BLT.26.010126

**Published:** 2026-01-01

**Gender-based violence**

Every year, from 25 November to 10 December, the 16 days of activism against gender-based violence call for global solidarity to end violence against women and girls. Violence against women is a major public health and human rights crisis, affecting almost one in three women in their lifetime. The risks increase in contexts of humanitarian crises, vulnerable settings and poverty. Yet, violence against women is preventable. Across the world, communities are challenging unequal gender norms, governments are strengthening policies and laws, and health systems are stepping up to provide care and support for survivors.

**Figure:**
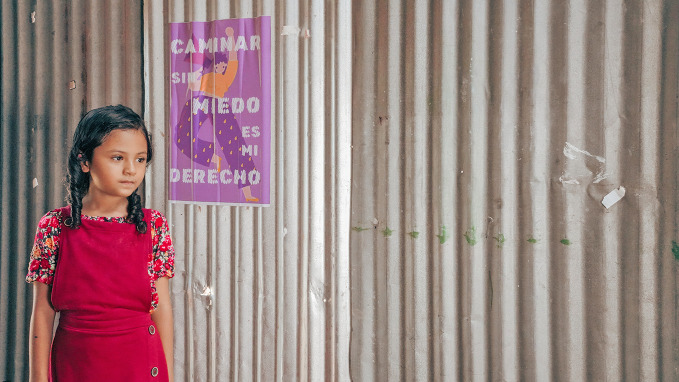
© UNICEF/UNI898869/Gonzalez Yepes

## Challenges to malaria elimination

New tools against malaria, including dual-ingredient mosquito nets and vaccines, helped avert an estimated 170 million cases and 1 million deaths in 2024, according the World Health Organization’s (WHO) *World malaria report 2025*. Since WHO approved the first malaria vaccines in 2021, 24 countries have introduced them into routine immunization programmes. Seasonal malaria chemoprevention has also expanded to 20 countries, reaching 54 million children in 2024.

Progress on elimination continues, with 47 countries and one territory now certified malaria-free. Cabo Verde and Egypt gained certification in 2024, followed by Georgia, Suriname and Timor-Leste in 2025. Still, 282 million cases and 610 000 deaths were recorded last year, 95% of them in the WHO African Region, mostly among children under five.

The report warns of mounting threats, including antimalarial drug resistance, widespread *pfhrp2* gene deletions that undermine rapid tests, expanding insecticide resistance and the spread of *Anopheles stephensi* to nine countries (Eritrea, Ethiopia, Ghana, Kenya, Niger, Nigeria, Somalia, Sudan and Yemen). Climate-driven extreme weather, conflict and stagnating funding are further straining efforts. 

“New tools for prevention of malaria are giving us new hope, but we still face significant challenges… none of these challenges is insurmountable. With the leadership of the most-affected countries and targeted investment, the vision of a malaria-free world remains achievable,” said WHO Director-General Tedros Adhanom Ghebreyesus.

https://bit.ly/4oEubeD

## Unified plan for coronavirus threats

WHO has launched its first unified strategic plan to help countries manage coronavirus disease threats, including COVID-19, MERS and emerging coronaviruses. The strategy marks a shift from emergency COVID-19 response to long-term, integrated management.

The *2025–2030 Strategic plan for coronavirus disease threat management *guides national authorities in adopting a coherent approach to surveillance, prevention and response within broader infectious-disease programmes. Coronaviruses have triggered repeated epidemics since SARS emerged in 2002. While the global impact of COVID-19 has declined, SARS-CoV-2 continues to circulate widely, causing severe disease in high-risk groups. 

“Coronaviruses remain one of the most consequential infectious disease threats today,” said Maria Van Kerkhove, WHO acting director for epidemic and pandemic management. While each country will have its own approach tailored to its national context, WHO urges Member States to use the strategic directions set out in the plan to build resilient health systems that can effectively manage current threats while preparing for future ones.” 

The plan covers both routine and emergency scenarios and was developed through extensive consultation. WHO has also expanded CoViNet, now comprising 45 reference laboratories across human, animal and environmental health sectors

https://bit.ly/48jgKf4

## GLP-1 therapies for obesity

WHO has released its first global guideline on the use of glucagon-like peptide-1 (GLP-1) therapies for treating obesity, a chronic and relapsing disease that affects more than 1 billion people worldwide. Obesity contributed to 3.7 million deaths in 2024 and global prevalence is projected to double by 2030.

Following the addition of GLP-1 medicines to the *WHO Essential medicines list* in 2025 for high-risk groups with type 2 diabetes, the new guideline issues conditional recommendations for their use as part of comprehensive obesity care. These therapies may be used by adults, excluding pregnant women, for long-term treatment, and may be combined with intensive behavioural interventions involving healthy diets and physical activity.

“Our new guidance recognizes that obesity is a chronic disease that can be treated with comprehensive and lifelong care,” said WHO Director-General Dr Tedros Adhanom Ghebreyesus. “While medication alone won’t solve this global health crisis, GLP-1 therapies can help millions overcome obesity and reduce its associated harms.”

The guideline stresses fair access, noting that GLP-1 therapies could reach fewer than 10% of those in need by 2030. WHO calls for action on affordability, manufacturing and health-system readiness to ensure equitable, person-centred care.

https://bit.ly/48IJgpA

## Lenacapavir rapid approval

The Zambia medicines regulatory authority has approved lenacapavir tablets and injectable formulations for HIV prevention in just 12 working days, marking one of the fastest national approvals on record. Shortly afterward, Zimbabwe’s medicines control authority authorized the product in 18 working days. Both approvals were achieved through the WHO listed authorities collaborative registration procedure (CRP), a reliance-based pathway that allows countries to make rapid, evidence-based decisions using assessments from trusted regulatory authorities or WHO prequalification.

These approvals highlight how regulatory innovation can accelerate access to next-generation HIV prevention tools. Lenacapavir, the first twice-yearly injectable pre-exposure prophylaxis (PrEP), provides a long-acting alternative to daily oral pills, helping people overcome challenges with adherence, stigma, or limited healthcare access.

“As the world marks World AIDS Day, these swift approvals in two African countries demonstrate how WHO’s collaborative registration procedure accelerates access to lifesaving medical products, while allowing national authorities to maintain full oversight and sovereignty over their decisions,” said Hiiti Silo, WHO unit head for regulation and safety.

WHO prequalified lenacapavir in October 2025, and applications in other countries through the CRP are ongoing, expanding access to this transformative HIV prevention option.

https://bit.ly/4iK6uQK

## Preventing sexual exploitation, abuse and harassment

42 African Member States, in partnership with WHO, have launched a landmark initiative to embed accountability for preventing and responding to sexual exploitation, abuse and harassment (PRSEAH) in joint health operations. 

This effort builds on WHO’s PRSEAH accountability framework for Member States, endorsed at the 78th World Health Assembly in May 2025. The framework provides a voluntary, adaptable starting point for ministries of health to institutionalize safeguarding in joint operations with WHO. It is aligned with United Nations system-wide standards but goes further by addressing sexual harassment alongside exploitation and abuse. 

The framework focuses on three mutually reinforcing areas: establishing clear policies and codes of conduct that set minimum standards for preventing and responding to sexual misconduct; equipping health personnel and partners with mandatory and specialized training, including modules for emergency responders and victim support teams; and ensuring robust incident management through safe reporting channels, survivor-centered assistance, as well as timely investigations backed by disciplinary or legal action. Together, these measures aim to protect communities and health workers during every intervention. 

https://bit.ly/44Kf7EP

## First guideline on infertility

WHO has published its first global *Guideline for the prevention, diagnosis and treatment of infertility*, calling on countries to make fertility care safer, fairer, and more affordable.

Infertility affects an estimated 1 in 6 people of reproductive age, yet access to care remains limited. In many countries, people pay for treatments out-of-pocket, with a single round of in vitro fertilization (IVF) sometimes costing twice the average annual household income.

“Infertility is one of the most overlooked public health challenges of our time and a major equity issue globally,” said WHO Director-General Tedros Adhanom Ghebreyesus. “Millions face this journey alone, priced out of care, pushed toward cheaper but unproven treatments, or forced to choose between their hopes of having children and their financial security.”

The guideline contains recommendations to strengthen prevention, diagnosis, and treatment, emphasizing cost-effective, evidence-based and people-centered care. It highlights the importance of addressing risk factors such as sexually transmitted infections and tobacco use, offering lifestyle guidance, clinical pathways from basic interventions to IVF and psychosocial support for affected individuals.

“The prevention and treatment of infertility must be grounded in gender equality and reproductive rights,” said Pascale Allotey, director of WHO’s Department of sexual, reproductive, maternal, child and adolescent health and ageing and the United Nations’ Special programme on human reproduction. “Empowering people to make informed choices about their reproductive lives is a health imperative and a matter of social justice.”

https://bit.ly/3KIWP03

**Cover photo**

Internally displaced persons in northern Gaza, occupied Palestinian territory, 2025.

**Figure:**
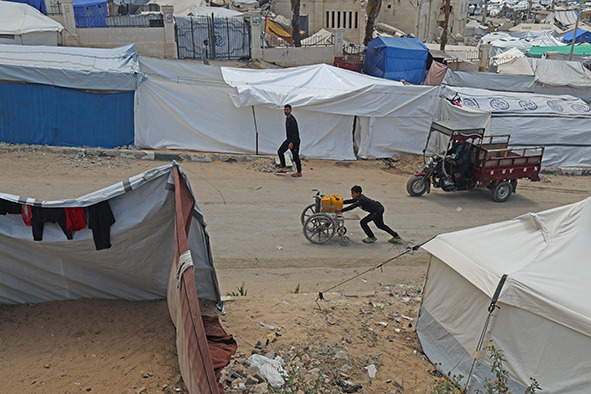
WHO

**Looking ahead**

3 March. World hearing day. https://bit.ly/4rDXHUl

9–12 March. Strategic Advisory Group of Experts on Immunization meeting. https://bit.ly/4rUGsOX

